# Supplementary material for: Chemosensory Proteins (CSPs) in the Cotton Bollworm Helicoverpa armigera
Source: Insects. 2021 Dec 27;13(1):29. doi: 10.3390/insects13010029 (PMC8780252; doi:10.3390/insects13010029)
Supplement: Supplementary file 1 [file insects-13-00029-s001.zip › Figures S2 and S3.pdf]

Supplementary data

S2

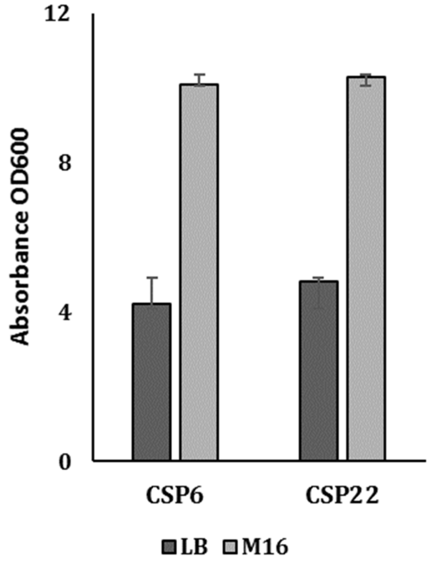

S3

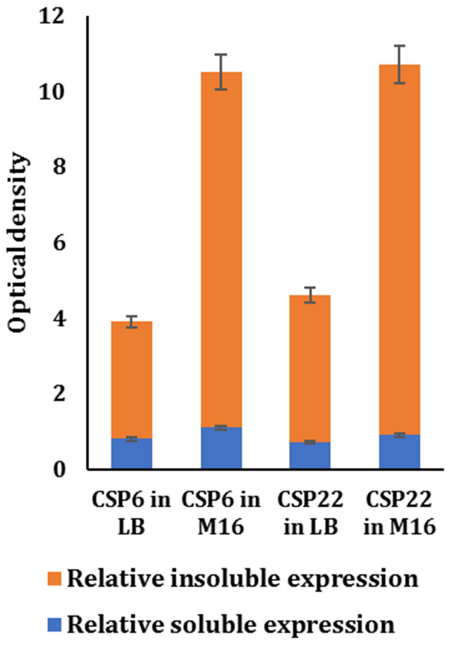

Figure S2: Chart showing level absorbance at 600 nm that represents the bacterial growth of *E. coli* cells expressing HarmCSP6 and HarmCSP22 in LB and M16 media.

Figure S3: Densiometric quantification of protein band showing relative soluble or insoluble expression of HarmCSP6 and HarmCSP22 in LB and M16 media.
