# Supplementary material for: Chemosensory Proteins (CSPs) in the Cotton Bollworm Helicoverpa armigera
Source: Insects. 2021 Dec 27;13(1):29. doi: 10.3390/insects13010029 (PMC8780252; doi:10.3390/insects13010029)
Supplement: Supplementary file 1 [file insects-13-00029-s001.zip › Supplementary Data 1.pdf]

>BmorCSP1
MKVLIVLSCVLVAVLADDKYTDKYDKINLQEILENKRLLESYMDCVLGKGKCTPEGKELK
DHLQEALETGCEKCTEAQEKGAETSIDYLIKNELEIWKELTAHFDPDGKWRKKYEDRAKA
KGIVIPE

>BmorCSP14
MKILIIVVMACVAVTWARPESTYTDKWDNINVDEILESNRLLKGYVDCLLGKGRCTPDGK
ALKETLPDALEHECVKCTGKQKSGADKVIRHLVNKRPDLWKELAVKYDPDNIYQARYKDK
IDAVKGSA

>BmorCSP8
MNSLIAFCLFAVLAVALARPDDKYTDRYDNVNLDEVLSNSRLLQPYIKCILDKDRCAPDA
KELKEHIREALETECAKCTEAQKKGTRRVIGHLINNESKSWNELTAKYDPENKFTAKYEK
ELREIKA

>BmorCSP9
MKTVIVCLLALTAVALARPEQYTDKYDTVDLDQLISNRRLLIPYVHCILEKGQCTAEGKE
LKSHIKEALETNCAKCTKAQKGGTEKMIGHLINHEAEFWEELKAKYDPTNEFTKKYETEL
KRVTA

>BmorCSP16
AHITDALQTGCTKCTGAQRKGIRRVIKHLIDSEPGYWDRLVDMYDPKRVYTGKYEKELRT
IKA

>BmorCSP6
MKCLTIAALLFVAGLSIAEKYTDKYDNIDVDEILENRKLLVPYIKCVLDEGRCTPDGKEL
KAHIKDGMQTACAKCTDKQKVSARKIVKHIKQHEADYWEQMKAKYDPKDEFKEIYEGFLA
GQN

>BmorCSP4
MFMLFIISFIIVPVLKCCGTETSTYTTQYDEVDIKEIMGNERLLVAYIGCLLDKNPCTPE
GKELKRNIPDALQSDCSKCSDKQRENADAWIEFMIDNRPEDWTKLEERYNPDGSYRTKYL
EGKHNATSNVDESK

>BmorCSP7
MKSVILICFLGVATVVIARPKTPFDNINIEEIFENRRLLLGYINCILERGNCTRAGKDLK
SSLKNVLEENCDKCSEDQRKSIIKVINYLVSSEPESWNQLKSKYDPEGKYLIKYEAKMES
N

>BmorCSP2
MKLLLVFLGLFLAVLAQDKYEPIDDSFDASEVLSNERLLKSYTKCLLNQGPCTAELKKIK
DKIPEALETHCAKCTDKQKQMAKQLAQGIKKTHPELWDEFITFYDPQGKYQTSFKDFLES

>BmorCSP3
MKSLIVLSCLLAACLAADLSKYENFDVEPIVTSDRLLKAYINCFLDKGRCTPEASDFKKA
LPDTIATNCGKCTEKQKANVRKVIKVIQQKHSTEWEKLVKKHDPSGKHRADFDKFLLGS

>BmorCSP15
MKLTSFLLVGMAMVSAEFYSSRYDDFDVKPLVENDRILQSYTNCFLDKGPCTPDAKEFKK
VIPEALETTCGKCSPKQKQLIKTVIKAVIERHPEAWEELVNKYDKDRKFRPSFDKFINED
D

>BmorCSP11
MKTILILCALVSVVVCRPEEYYSSQYDNFDVEQLVGNLRLLKNYAKCFLDQGPCTAEGTE
FKKRIPEALRTKCAKCNPKQRHLIRTVVKAFQTKLPDLWEELAIKEDPKGQYKHEFTAFI
NAMD

>BmorCSP13
MKFVLALIALAVVVAARPNDDLFYDKKYDNFNVDEIIDNPRLLKAYTFCFNDKGKCTAEG
NDFKKWIPESLQTSCGKCSEKQKYLVAKFVHAIKDKMPDEFDILRKLHDPKGEYTENLDK
FLETYGH

>BmorCSP17
MKSSLFCVLVLTVVVSSSRQQSYPRNDNININAILQNDRILLGYFKCVMDRGPCTKDGKT
FKRALPEALPTACARCSNKQKAAFRTLLLAIRARSEPSFLELLDKYDPSRSNRELLYTFL
ATGL

>BmorCSP10
MRAVIFLYTCVFVVVGQDINAMMSMPKYDERYDYLDVDDIFRNKRLVRNYVDCLINAQRC
TPEGKALKRILPEALRTKCIRCTERQKRTSVKVIRRLKNEYPEEWAKLASRWDPTGDFTR
YFEDYLAKEHFNTIPGSGL

>BmorCSP18
MNNLLIAILALTLPFSIWCYDEKYDKIDVDKILSDDKLFTDYINCMLDKGPCEVEYSSEF
KELLPEVIATSCAKCTPIQKTGLRKTVKALSVKRPDDFSQFRAKYDPKGEYEKQFAAFVV
ATD

>BmorCSP22
MNNLLIAILALTLPFSIWCYDEKYDKIDVDKILSDDKLFTDYINCMLDKGPCEVEYSSEF
K

>BmorCSP5
MKFLVTAVLLSLAIAIQAGSYSDRYDNINVAEILGNKRLLTAYIKCVLEEGKCTAEGKEL
K

>BmorCSP12

MKGFYVLCFALFAAVYCKETYSSENDDLDIEALVGNIDSLKAFIGCFLETSPCDAVSGDF
KKDIPEAVAEACGKCTPAQKHLFKRFLEVVKDKLPQEYEAFKTKYDPQGKHFDALLSAVA
NS

>BmorCSP19
MIENFYSKCTISKSVLFLCLIFLPYALNQKYYDSRYDYYDIDHLVQNPRLLKKYLDCFLG
KGPCTPIGRLFKQVMPEVITTACAKCTPTQKRFARKTFNAFRRYFPETLMELRRKFDPES
KYYDAFEKVITNA

>BmorCSP20
MIEWKRFKILHFLSYLGLLVLVVVCAAQQNRPQVTDTALDEALNDKRFIQRQLKCALGEA
PCDPIGKRLKTLAPLVLRGACPQCSPQETKQIQKTLSYVQRNFPQHWAKLVRQYAG

>BmorCSP21
MPPNLKNIVFIGVCTCLVLTVLAAPQMSDAQLEKTLADKGTMQRHLRCALGEGPCDMVGR
RLRTLAPFVLRGACPQCSVQESRHIRRTLAYIQRNYPWEWARIVRQYG

>HarmCSP1
MKFVLVLCLFAAAALADDKYTDKYDNINLDEILENKRLLLAYVNCVMERGKCSPEGKELK
EHLQDAIETGCSKCTEAQEKGAYKVIEHLIKNELDIWRELAAKYDPKGDWRKKYEDRARA
NGIQIPE

>HarmCSP2
MKVVLLTLCFALGVLAQDQYESANDNFDISEVIGNDRLLHAYANCLLNKGPCTPEVKQVK
EKLPEALETRCAKCTDKQKQMGKALAQEVKKNHPDIWKQLVAMYDPQGKYQQAWKDFLQE

>HarmCSP3
MKVLVVLSCLIVAAFAADKYNAKYDNFDVDTLITNDRLLKAYINCFLDKGRCTPEGSDFK
KTLPEAIETTCGKCTDKQKNNIRKVIKAIQQKHPKEWDALVKKNDPSGKHRANFDKFIQG
SR

>HarmCSP4
MMLLVYLTIQSNAVETSTYTTKYDGIDLDEILNNERLLTGYVNCLMDNGPCTADGKELKR
NLPDAIENDCKKCTDRQREGADRVMHYLIDHRPEDWTKLEKKYNSDGSYKMKYLSRKPAE
DSKETNTTKSEEDTKNETKEST

>HarmCSP5
MKFFVVAIVVALAAFAVAETYTDRYDNINIDEIIENRKLLVPYIKCVLEQGRCTPEGREL
KAHIKDALQTSCTKCTQKQRKASRKVVKHIRANELDYWKQLLAKYDPDSIYEKNYESFLA
ADD

>HarmCSP6

MKVFVVLSVLIAFTAAASLTPAELDLAEAFDYEALFSNDEQRKLVFDCILGKAECGDYQK
MAEISRKVLESKCADCNASQKAKYEIVLKTIQTKYEPFYNELLKNVAAKKE

>HarmCSP7
MQVVVFLSVICVGLVAGLHVQAGPQMTDAQLDQTLADKNTMQRHIKCALGEGPCDPVGRR
LRTLAPLVLRGACPQCSMQETRQIRRTLAFVQRNYPWEWAKIVRQYG

>HarmCSP8
MWTQVITFSVALTIALGEEVVGGIKREVSEGVQSMGYKLIYGDEDMTIINQVVTDAEKSD
MVKKNKVNLNEAIKPLAPEDVKCLMSVDRYCSKKMGAMKSVLIQAVKEDCAKCSIKQKDE
AGKVIASMMAHDPVAWKLFLTRYDGLDKVQRILG

>HarmCSP9
MRTFVVVCLLGLVAVTLARPESKYTSKYDNINLDEILANQRLLVPYLKCILEEGKCTPEG
KELKSHIREALEEDCAKCTENQRKGTRKVLAHLINHEEGYWNRLKAKYDPESKYTAKHEQ
ELRELKH

>HarmCSP10
MKVLVLLLAAVVTAQYEEDTYGTDHDDLDIAAVVEDKDQFNSFVDCFIDEAPCDDVAETF
KSVIPEAVLEACAKCTPAQKHIVRVFNESFKKKMPEKFQKFKNKYDPEGKYFDNFEAAVA

AF
>HarmCSP11
MKSLLILCLVIAAVWARPETYDTRYDDFDAETLVENVRLLKAYGHCFLGTGPCTPEGSDF
KKTIPDALRTGCGKCTAKQRHLIRVVVQGFRSKTPDLWQQLVKKEDPNGQYKEIFTRFLN
GSD

>HarmCSP12
MKCIYVLSFLLALAAVQAEDKYSTENDNLDIDAVVANVDTLTNFVACFVDQEPCDAVAAD
FKKDIQEAVTTRCAKCTDAQKHIFYKFILGLKEELPRGYEEFRRKYDPENKHFSALENAV
SPA

>HarmCSP13
MKLLIVLALVAVVAARPDDEFYDKKYDDFNVDEIIENVRLLKAYAHCIIGDGKCTPEGND
FKRWVPEATKSSCGKCTEKQKVLVAKTIKAIKEKCPEEYTTLIKQLDPENKYADDLKNYL
AKYGH

>HarmCSP14
MKLIVAVALLCLVAESWAASTYTDKWDNINVDEILESQRLLKAYVDCLLDRGRCTPDGKA
LKETLPDALENECSKCTDKQKSGSDKVIRHLVNKRPEMWKELSAKYDPNNIYQDRYKDKI
EAVKGQ

>HarmCSP15

MKADCFLIITLVAVVAADFYNSKYDSFDVQPLLENDRILLSYTKCFLDQGPCTPDAKDFK
KVIPEALETTCGKCSPKQKQLIKTVIKAVISRHPDAWDQLTEKYDKDKKYKESFDKFLAE
QD

>HarmCSP16
MQTRYAVVLCCVVAACVAQTQRPPVSDSALEDALQDKRFIQRQLKCALGEAPCDPIGKRL
KTLAPLVLRGACPQCTPQETKQIQRTLSYVQRNFPQQWAKIVRQYAG

>HarmCSP17
MRSWLICLCVLTVVVTCHSQAPNRYENFNADAIIQNDRILLAYYKCVMDKGPCTRDGKNF
KRVLPETLATACGRCNPKQKTIVRKLLLGIRSKSEPRFLELLDKYNPDRSNRDALYAFLV
TGN

>HarmCSP18
MKVLMVAVLALVAPSALGYDEKYDKLDVDKIIGDDALFTAYIDCMLDKGPCTVEHSEDFK
KLLPEVIQTACAKCSGIQRTNVRKTVKALSDKKPDDFAKFRAKFDPKGEYEKDFSAFMLG
TD

>HarmCSP19
MIDLNKCNLKVSILILCLYFVSINAQQRFYDRRYDYYDIDTLIQNPRLLKKYLDCFLEKG
PCTPIGRVFRQILPDAVATACEKCSPSQRRLARKTFNAIRRNFPQGYVELMSKLDPKNKY
YEAFGKAIANA

>HarmCSP20
MKAVFLLCLVVVAVSARPEAQYTNKYDNVNLDEILVNKRLLVPYIKCALDQGKCSPDGRE
LKSHIREALENYCAKCTPVQQDGTRRVIAHLINHEPDYWRQLSVKYDRDGKFAVKYEKEL
RTIA

>HarmCSP21
MNSAIVLCVVALAGMVLARPDGGTYTTKYDNVDLDEILANDRLLIPYIKCLLDEGKCAPD
AKELKEHIREALENGCAKCTDKQKEGTRRVIAHLIKHKNADWQKLKAKYDPEGKYTHKYE
KELEEVQH

>HarmCSP22
MNSAIVLCVVALAGMVLARPDGGTYTTKYDNVDLDEILANDRLLIPYIKCLLDEGKCAPD
AKELKEHIREALENGCAKCTDKQKEGTRRVIAHLIKHKNADWQKLKAKYDPEGKYTHKYE
KELEEVQH

>HarmCSP23
MNSAIVLCVVALAGMVLARPDDTYTTKYDNVDLDEILGNDRLLVPYIKCTLDEGKCAPDA
KELKEHIREALENGCAKCTDKQKEGTRRVIAHLIKHKLAEWEKLKAKYDPEGKYTHKYEK
ELEEVKNA

>HarmCSP24
MNSAIVLCVVALAGMVLARPDGDGDKYTSKWDNIDLDEILGNDRLLVPYIKCTLDEGKCA
PDAKELKEHILEALETGCDKCTDKQKEGTHRVIAHLIKYKLEEWEKLRAKYDPEGKYAKK
YEKELEELKKA

>HarmCSP25
MNSAIVLCVVALAGMVLARPDGDGDKYTSRWDDVDLDEILENDHLLVPYIKCSLDEGKCA
PDAKELKEHIQEALETGCAKCTDKQKEGTRRVIAHLIKKKLQEWEKLKAKYDPEGKYAKK
YEKELEEVKNA

>HarmCSP26
MNSLIVFCVLSLAALTIARPDGATYTDKYDNVDLDEILGNRRLMVPYIKCMLDQGKCAPD
AKELKEHIKEALENECGKCTEAQKKGTRRVIGHLINHEADFWNELTAKYDPERKYTTKYE
KELKEVKA

>HarmCSP27
MNSLIVFCVLSLAALTIARPDGATYTDKYDNVDLDEVLSNRRLLVPYVHCLLEQGKCAPD
AKELKEHIREALENACGKCTNAQQSGTRRVIGHLINKEPEFWKQLNAKYDPNNKYTKKYE
KELKEVQEEHH

>HvirCSP1
MKFLLVLCVMAAVALADDKYTDKYDNINLDEILENKRLLLAYVNCVMERGKCSPEGKELK
EHLQDAIETGCSKCTEAQEKGAYKVIEHLIKNELDIWRELTAKYDPKGDWRKKYEDRARA
NGIQIPE

>HvirCSP2
MKVVLITLCFALGVLAQDQYESANDNFDISEVLTNDRLLQAYANCLLNKGPCTPEVKQVK
EKLPEALETRCAKCTDKQKQMGKTLAQEVKKNHPDIWKQLVSMYDPQGKYQQAWKDFLQE

>HvirCSP3
MKVLVVLSCLVVAAFAADKYNAKYDNFDVDTLITNDRLLRAYINCFLDKGRCTPEGSDFK
KTLPEAIETTCGKCTDKQKNNIRKVIKAIQQKHPKEWDDLVKKNDPSGKHRANFDKFIQG
SR

>HvirCSP4
MMLLVYLTIQSNAVETSTYTTKYDGIDLDEILNNERLLTGYVNCLMDNGPCTADGKELKK
NLPDAIENDCKKCTDRQREGADRVMHYLIDHRPDDWTKLEKKYNSDGSYKMKYLSRKTED
SKETNTTNSEEDTKNETKEST

>HvirCSP5
MKFLVVAILVTLAAFVVAETYTDRYDNIDIDEIIANRKLLVPYIKCILDQGRCTPEGKEL
KAHIKDALQTSCTKCTDKQKKASRKVVKHIRAKEQDYWKQLLAKYDPDSQYVQNYESFLA

ADD

>HvirCSP6
MKVFVVLSVLIAFTAAASLTPAELDMAEAFDYDALFSNDEQRTLVFNCMLGKGDCGDYQK
VADISRQILETKCADCTPSQKAKYEKVLKTLQTKYGDFYTELLKNVKKE

>HvirCSP7
MQVVVFLSVICVGLVAGLHVQAGPQMTDAQLDQTLADKNTMQRHIKCALGEGPCDPVGRR
LRTLAPLVLRGACPQCSMQETRQIRRTLAFVQRNYPWEWAKIVRQYG

>HvirCSP8
MGYKLIYGDEDMTVINQVVTDAEKSSMIKKNRVNLNEAIKPLAPEDVKCLMSVDRYCSKK
MGAMKSVLIQAIKEDCAKCSIKQKDEAGKVIASMMAHDPVAWKLFLTRYDGLDKVQRILG

>HvirCSP9
MRTFIVLCFLGLVALTLARPDTKYTNKYDNINLDEILANQRLLVPYLKCILEEGKCTPEG
KELKSHIREALEEDCAKCTDNQRKGTRKVLAHLINHEEGYWNRLKAKYDPESKYTSKHEQ
ELRELKQ

>HvirCSP10
MKILVLLFAAVVTAQYEETYGTDHDDLDVVAVVSDKEQFNSFIDCFIDEGPCDEIAATFK
SVIPEAVLEACAKCTPAQKHLVRVFNENLKKKMPEKYQKYKNKYDPEGKYFDNFEAAVAS
F

>HvirCSP11
MKSLLVLCLLLAAVCARPETYDTRYDDFDAETLVENVRLLKAYGHCFLGTGPCTPEGSDF
KKTIPDALKTGCGKCSGKQRHLIRVVVKGFQSKTPDLWQQLVKKEDPNGQYKETFTKFLN
GSD

>HvirCSP12
MKCVYVLSFLLAVAAVHAEDKYSTENDDLDIDAVVANLASLREFVGCFMDAVTCNAIAAD
FKKDIPEAVSTQCAKCTDAQKHIFYKFLLGLKEKLPNDYQAFKIKYDPDNKHFSALEDAV
SGA

>HvirCSP13
MKLFIVLALVAAVVGRPDEAFYDKKYDDFNVDEIIDNVRLLKAYAHCIIGDGKCTPEGND
FKKWVPEATKSSCGKCTEKQKVLVAKTIKAMRDKLPEEYTTLVKQIDPENKYDEDLKSYL
AKYGS

>HvirCSP14
MKFIVAAALLCLVAESWAASTYTDKWDNINVDEILESQRLLKAYVDCLLDRGRCTPDGKA
LKETLPDALENECSKCTEKQKAGSDKVIRYLVNKRQDLWKELSAKYDPNNIYQDRYKDKI
EAVKGQ

>HvirCSP15
MKADCLLIVTLVAVVAADFYNSKYDSFDVQPLLENDRILLSYTKCFLDQGPCTPDAKDFK
KVIPEALETTCGKCSPKQKQLIKTVIKAVINRHPDAWNQLIEKYDKDKNYKESFDKFLAE
QD

>HvirCSP16
MQTRYALVLCCVVAACVAQTQRPPVSDSALEDALQDKRFIQRQLKCALGEAPCDPIGKRL
KTLAPLVLRGACPQCTPQETKQIQRTLSYVQRNFPQQWAKIVRQYAG

>HvirCSP17
MRSWLICLCVLTVVVSCYSQAPNRYENFNADAIIQNDRILLAYYKCVMDKGPCTRDGKNF
KRVLPETLATACGRCNPKQKTIVRKLLLGIRSKSEPRFLELLDKYNPDRSNRDALYAFLV
TGN

>HvirCSP18
MKVLVVAVLALVAPYVLAYDEKYDKLNVDKIIGDDALFTAYTDCMLDKGPCTVEHSADFK
QLLPEVISTACAKCTAIQRQNVRKTVKALSEKKPDDFTKFRAKFDPNGEYEKAFSAFMLA
TD

>HvirCSP19
MIDLNKCNFRLSISILCLFFVSINAQRYYDRRYDYYDIDTLIQNPRLLKKYLDCFLWKGP
CTPIGRVFRQILPDAVETACEKCTPSQRRLARKTFNAIRRNFPEGYVELMKRLDPKNKYY
EAFEKAIANA

>HvirCSP20
MKAVFLLCLVVVAVSARPESQYTNKYDNVNLDEILVNKRLLVPYIKCALDQGKCSPDGKE
LKSHIREALENYCAKCTPVQQDGTRRVIAHLINNEPDYWTQLSAKYDRDGKFALKYEKEL
RTIA

>HvirCSP21
MNSAIVLCVVAVAGMVLARPDGDTYTTKYDNVNLDEILANDRLLVPYIKCLLDEGKCAPD
AKELKEHIREALETGCAKCTDAQKNGTRRVIGHLIKHKNEEWQKLKAKYDPEGKYTHKYE
KELDEVQAGR

>HvirCSP22
MNSPIVLCLVALAGMVLARPDGDGDKYTSKWDNVDLDEILANDRLLVPYIKCVLDEGKCA
PDAKELKEHIREALETGCAKCTDAQKDGTRRVIAHLIKKKVEEWEKLKAKYDPEGKYSQK
YEKELEEVKNA

>HvirCSP23
MNSAIVLCVVALAGMVLARPDGDGDKYTSKWDNVDLDEILANDRLLVPYIKCVLDEGKCA
PDAKELKEHIREALETGCAKCTDAQKDGTRRVIAHLIKKKIEEWEKLKAKYDPEGKYSQK
YEKELEEVKNS

>HvirCSP24
MNSAIVLCVVALAGMVLARPDGDGDKYTSKWDNVDLDEILANDRLLVPYIKCVLDEGKCA
PDAKELKEHIREALETGCAKCTDAQKDGTRRVIAHLIKKKVEEWEKLKAKYDPEGKYSQK
YEKELEEVKNA

>HvirCSP25
MNSAIVLCVVALAGMVLARPDGDGDKYTSKWDNVDLDEILANDRLLVPYIKCVLDEGKCA
PDAKELKEHIKEALETGCAKCTDAQKDGTRRVIAHLIKKKVEEWEKLKAKYDPEGKYSQK
YEKELEEVKNA

>HvirCSP26
MNSLIVFCVLSVAAMALARPDGAAYTDKYDNVDLDEILSNRRLLVPYVKCILDQGKCAPD
AKELKEHIIEALENECGKCTEAQKKGTRRVIGHLINNEADYWNELTAKFDPEKKYVQKYE
KELKEVKA

>HvirCSP27
MNSLIVFCVLSAAAMALARPDTYTDKYDSVDLDEVLSNRRLLVPYVNCLLEQGKCAPDAK
ELKEHIREALENACGKCTEAQKNGTRRVIGHLINKEPEFWNQLKAKYDPNNKYTQKYEKE
LKEVQEDKQ

>HvirCSP28
MNSAIVLCVVALAGMVLARPDGDGDKYTSKWDNVDLDEILANDRLLVPYIKCVLDEGKCA
PDAKELKEHIKEALETGCAKCTDAQKDGTRRVIAHLIKKKVEEWEKLKAKYDPEGKYSQK
YEKELEE

>HvirCSP29
MNSLIVFCVLSVAAMALARPDGAAYTDKYDNVDLDEILSNRRLLVPYVKCILDQGKCAPD
AKELKEHIREALENECGKCTEAQKKGTRRVIGHLINNEADYWNELTVKYDPEKKYVQKYE
KELKEVKA

>MsexCSP1
MKCICLLFLVVVAVYAEEKYTEENDDLDIEGVIKDADTMKAFTGCFMDTADCDHVSGDFK
KDLPEAIQTACAKCTDKQKHITKRYFEGLEEKYPELYQAFKNKYDPENKYFAALKAAIAK
F

>MsexCSP2
MKVLLLAVVFVATVWCYDEKYDKIDVDKIIADDNLFKAYIDCMLDKGPCKEEYSEDFKKL
LPEVIATSCSKCNEIQRQNVRKTVKALSNKKPDEFNEFRKKYDPNREHEKDFAAFVLAVD

>MsexCSP3
MKTIVVCLFALFAVVLAKPKEFYTDRWDSVDIDSILANRRLLNPYILCILEEGKCTPEGK

ELKSHIRDAMQTDCAKCTPVQKAATERVIAHLLKHEHESWNKLTAKYDPTGAYTKAHHDQ
LKSLAA

>MsexCSP4
MQVTYVLLVCVVVASCVAQQAQRPQVTDTALEDALNDKRFIQRQLKCALGEAPCDPIGKR
LKTLAPLVLRGACPQCSPQETKQIQRTLSYVQRNYPQQWAKIVRQYAG

>MsexCSP5
MKTLVFFLCLVLAVLADEKYETINEDFDVAQVLENERLLNSYAKCLLNKGPCTPEVKKVK
DKLPEALETHCAKCTDRQKAMGKQLAQEVQKRYPDLWKELVALYDPEGKYQDAFKEFLAN

>MsexCSP6
MMKWQIAIALMVVVAVVSCDEKYTTKYDNINYKEILENKPLLHNYIKCTLDKGRCTAEGN
ELKSKIKDALQTGCIKCSDKQKQGARDVIQHLEKHEPEYFAELRAKYDPNNEFESTMRDF
LAGKI

>MsexCSP7
MKTILVLFALVAAVACEEYYSTQYDNFDANELVSNVRLLKNYGKCFLDEGPCTVEGREFK
KNIPDALRTRCRKCTPKQRHLIRTVVKGFQTKLPDLWERLAKKEDPEGIYKEDFLAFINS
TD

>MsexCSP8
MKTWLLCLVLTVVVSRSTQQNYPRNENININAILQNDRILLGYYKCVMDKGPCTKDGKVF
KRALPEALPTACARCSQKQKAVFRTLLLAIRAKSEPRFMELLDKYDPSRANRDTLYKFLA
TGV
>MsexCSP9
MQNFIVFCLCICILPLAKSAPQMTDAQLEQTLNDKATMQRHLKCALGEGPCDPVGRRLRT
LAPLVLRGACPQCSPQETRHIRRTLAYVQRNYPWEWARIVTYALLVCVVVASCVAQQAQR
PQVTDTALEDALNDKRFIQRQLKCALGEAPCDPIGKRLKTLAPLVLRGACPQCSPQETKQ
IQRTLSYVQRNYPQQWAKIVRQYAG

>MsexCSP10
MQILLTHAEENSTYTTEYDGFDIREVMRNERLLTSYVNCLLDKGPCTAEGKELKKNLPDA
AQNDCKKCTQRQKENADLMIQYMEENRPADWNKLELNTSGERVAGASERHISVNTKRALF
HT

>MsexCSP11
NLNRQRKYFRTFLKTMEAKCTLFILLVAMVSADFYSSKYDDFDVQPLLENDRILLSYTKC
FLDEGPCTPDAKDFKKVIPEALETSCGKCTPKQKKLIKQVIRAVIDRHPESWDKLVHKYD
EDNKYKDSFNKFLAEKD

>MsexCSP12
MRLLVIMSLFALSLVAARPDASKYPSRYDTVDLDMIVNNKKVLESYLKCVLDEGKCTPEG

KELKAHIKEALETYCAKCTEPQKEGTRFVIGHLVKNEKEWWRKLSDKYDPERKYVTKYEA
ELKSIS

>MsexCSP13
MRTVIVLTFLVAACFAAEKYNPKYDNFDVDTLISNERLLKAYINCFLDKGRCTPEGTDFK
KALPEAVETTCAKCTEKQKVNIRKVIKAIQQKYPKQWEELVKKNDPSGKHRANFDKFIQG
S

>MsexCSP14
MLDTMNAIRVLVFCVCMYVVVGQDINQMANMPKYDSRYDYLDVDAIFTNKRLVRNYVDCL
INAQRCSPEGKALKRILPEALRTKCIRCTERQKITAVKIIERLKYEYPEEWAKLSSRWDP
TGDFTRYFEEFLAKESFNTIPGSGSTVNEVSVTTATP

>MsexCSP15
MRLLVIMSLFALSLVAARPDASKYPSRYDTVDLDMIVNNKKVLESYLKCVLDEGKCTPEG
KELKAHIKEALETYCAKCTEPQKEGTRFVIGHLVKNEKEWWRKLSDKYDPERKYVTKYEA
ELKSIS

>MsexCSP16
MTTMKMLAVLCLFVLGALSAPERDGDLYDMFDAKMILEDDKLRSKAIDCLLDRGVCDDYQ
PIRDKGPRLIKTRCEDCTPEQKAVFEESMKILEEKFNNDFKEIIAKYA

>MsexCSP17
MKTFVALCLLSVVAVTLARPDHYTDRYDNVNLDEILDNHRVLVPYIKCILDQGKCAPDAK
ELKEHIREALETECSKCTNAQKNGTRRVIQHLINHEPEYWQELGDKYDPERKYTVKYEKE
LREIKA

>MsexCSP18
MKYLLVLCCVVAAVVCDDKYTDKYDNVNVDEILANERLLKGYVDCVLERGKCTPEGKELK
EHLRDAIETGCKKCTKPQEEGATKVIDFLIKNKLEVWRELVAKFDPEGKWRKKYEDRARA
NGIVIPE

>MsexCSP19
MKPVTAAILISLACMVQCGKDMYTSRYDSMNVDDVIGNHRLLHAYIKCMLDEGRCTAEGR
ELKKHITDALQTGCSRCTDAQKKAIRHVIKHLIEHEHDFWALLVEKYDPHRIYTTKYEAE
MKRTMRSKEQMSESGAGHEKADMKMMGDGPGYKKADMKMMEGGSGHEKADMKMMEGGPGH
EKADMKMMEGGPGHEKADMKMMEGGPGHEKADMKMMDKMSSKTGMAEKKGA

>MsexCSP20
MKMLLLVISCCVALPWALSASTYTDKWDNINVDEILESDRLMKGYVDCLLDKGRCTPDGK
ALKETLPDALEHDCSKCTEKQKVGSEKVIRNLVNKRPALWKELSAKYDPNNLYQEKYKDK
IDSIKGQ

>HmelCSP1
MKCVLLLGFLVFSIALAEKYSDKYDDFDIDEVINNKRLMVSYLKCFLDQGRCTPEGKDVK
TYIKDAMETGCEKCTEAQRKKARVVVSHVRTNEKEYWEEMKKKYDPNSEYKEVYEAFLAR
DD

>HmelCSP2
MKFVILCVFALIAVASSRPESKYTDRFDNIDTQQILENRRLLVAYILCILEKGKCTNEGR
ELKGTLKTLLETECSKCTEVQRKGTRTVIGHLINHEEDYWNQLVVKYDPDRKYVVKYEKE
LRSVKA

>HmelCSP3
MKTVLCLFALVAVACALPSSTYTDKYDNIDLDEILSNKRLLTPYVKCMLDEGKCSPEGKE
LKSHVREALENECSKCTENQKAGSRKVIGHLINNEQEYWSKLTAKYDPDQKYAKKYEKEL
KTVSA

>HmelCSP4
MVSKTFVVLCCFVALAWAGEKYSDKYDNIDLEEIMENDRLLNSYSNCMLDKGPCTPEGKE
LRDHMEDALKNGCEMCTETQKDKSTTMIHFLINKKLDIWKQLTAKYDPEGVWRTKYEDEA
KKRGIEIPKE

>HmelCSP5
CAITVNARPAEHYTDKYDGIEIKEIINNRRLLLPYLKCLLDQGKCSPEGKELKSHIQEAL
ETYCAKCTEAQRKGTRQVIAHLINNEGDYWNELTAKYDPKKQYVKKYENELKTVKS

>HmelCSP6
MKFLIVLSCLVLSAFAGEKYNAKYDNFDVETLVTNERLLKSYINCFLDKGRCTAEGSDFK
KTLPEAVETVCGKCTEKQKINIKKVIRAIQEKFPKYWEELVQKNDPSGKHRENFDKFIKS

>HmelCSP7
MKCLLVLSGLLVLAAAQQYSSENDDLDIEAVVKDPETMKVFAECFNDKGSCNEQMADFKK
DIPEAVVEACAKCTPAQKHIFRRFLEVLKVKNPQEYQYFKQKYDPQDKHFAALEAAIANA

>HmelCSP8
MKTILVLATVLAAAVAAPADTYNPEYDNFNAEEIVENVRLLKNYAKCFLDEGPCTAEGTD
FKKTIPEALKTNCAKCTPKQRELIRTMVRGFQTKLPDLWKELAMKQDPNGEYKESFEKFL
NSTD

>HmelCSP9
MKVIVIMLCLCVTVLSQDIESMRNMPKYDSRYDYLDVDALFNSKRLVRNYVDCLINGQRC
TPEGKALK

>HmelCSP10
MKSVILVALMCLVAAAWGKPASTYTDKWDNINIDEILESNRLLKGYVDCLLDRGRCTSDA

KTLKETLPDALEHDCNKCTAKQKSGSDKVISHLVNKRPELWKELSVKYDPNNIYQEKYKD
KLQTIKA

>HmelCSP11
MKIFIAFIIALPSVLTDFYSSRYDDFDIQPLLENDRILLGYTRCFLDQGPCTPEAKDFKK
AIPEAVETTCGKCTPKQKELIRQVIRAVMQSHPDAWDQLVDKYDKDKKYRQNFDNFIKDD

>HmelCSP12
MHIIQQSVLILLLVYHYDCETYTTKYDGVDLDEILASDRLLTGYVKCLLDNGPCTPDGKE
LKKNLPDAIDNDCRKCTEKQRIGADKVMHYIIDHRPDDWDKLEKK

>HmelCSP13
MKIILLFCAIGIALVFAEEKYDSSNDNIDLSEVLGNDRLLTSYSKCLINKGPCTPEVKKL
KDRLPEALDTGCAKCTEKQKQMGRQLVKEVKEKHPQIWKELVAHYDPQGKYRQAFEEFLK
A

>HmelCSP14
MKIVIVLALIAVVAARPADDYSRYDNFDINEIIDNLRLLKSYNHCFLGTGKCTPEGSDFK
KWIPDAVQTSCGKCSDNQKVLVAKVIDATMKKLPEDWEKLNMAHNPDGKYDTTLKEFLDK
YGN

>HmelCSP15
MRKVSIIIILLISCVLTQERFYDRRYDYYDIDQFVENPRLLKKYLDCFLDRGPCTPIGRVF
KAVLPEIVRTACSKCSPSQKRFARRTFEAFQRYSPESHSELKKKLDPQNKYFTTFETVLS
RS

>HmelCSP16
CMAQTQRPEVSDTALDDALNDKRFIQRQLKCALGEGPCDPIGKRLKTLAPLVLRGACPQC
TPQETNQIQRTLSYVQRNYPQQWTKIVRQYSG

>HmelCSP17
MNNWLLYLCALTVVGSCAAQQYQQFVNKYDNFNADSIIQNDRILLAYYKCVMDKGPCTKD
GKNFKRALPETLTTACGRCSPKQKAVVRKLLLGIKSKSEPRFVELLDKYDPTQANRAALY
SFLVTGV

>HmelCSP18
MLVPYMILLVTVAYTVECYDEKFDKLDVDKVINDETLFNSYLNCFLDKGPCTAEYASEFK
DILPELLSDACAKCSKLQKNNVRRVFKEMYEKRTDALAEFEKKYDPKGENKANFLSILQS
KED

>HmelCSP19
MKTILVLAATLAVALAHPADTYNPIYDNFNAQELVENVRLLRNYGKCFLYQGPCTAEGMD
FRRAIPDALKTNCGRCSPKQRQLIRDVVRGFQAKLPAIWQELTEKEDPKGEYKESFEQFL

NSSD

>HmelCSP20
MQLLLAAATLVASLIITKAQVNSERLDSVNIDEVLANKRLLQAYIKCTLDKGRCTPEGRE
LKTHITEALQQGCDGCTEGQKESVRRVIRHLIRNEPDYWQQLVDKYDPELVFSKKYEDEL
NSL

>HmelCSP21
IIISICVTVMAAPQMSDIQLERTLADRGTMQRHIKCALSEGPCDPVGIRLRTLAPLVLRG
SCPQCSAKETHQIRRTLAFVQRNYPWEWAKIVRQYG

>HmelCSP22
VAKKSIVVICWFLASVFAVEKYTDKYDNINMQEILDNRRLLLAYANCILDKGRCSPEGKE
LKEHIQDALETGCKKCTEAQINGATVVIEHLIKKEGDIWKQLTDKYDPQGRWRKKYEDRA
REKGIVIPQD

>HmelCSP23
MVAKSIVLVCCFLASVLAVEKYTDKYDNINIQEILDNKRLLIAYANCLLDKGKCSPEGKE
LKDHIQDALETGCEKCTEAQVNGTTTIIDHLIKNERSIWKELTEKYDPKGIWRKKYEDRA
REKGIVIPED

>HmelCSP24
MASKLIVLMCCFVATVLAVDKYTDEFDNIDIQEILDNKRLLRSYANCILDKGKCTPEGKE
LKEHLQDALETGCEKCTDVQGKETRRVIKHLIKQELEIWKELTDKFDPKGIWRKKYEEQA
KAEGIIVPQD

>HmelCSP25
MAVLIFTILFLISSISSEMIVAGIDRSVSDGVRSMGYNVVYGDEDLMVINEVVRDSEKYN
SWKAKTDLNHSIPPLRAEDVKCLMSVDRYCSKEMGEMKNILIQALKDDCTKCTTKEKESA
GRTVASMMAHDPVAWKLFLTKYDSIGK

>HmelCSP26
MASKLIVLMCCFVATVLAVDKYTDEFDNIDIQEILDNKRLLRSYANCILDKGKCTPEGKE
LKDHLQDALETGCEKCTNVQEKETKRVIKHLIKQELEIWKELTDKFDPKGIWRKKYEEQA
KAEGIIVPEM

>HmelCSP27
MASKLIVLLCCFVAVALAVDKYTDKFDNLDIQEILDNKRLLRSYANCLLDKGKCTPEGKE
MKEHVQDAIETGCEKCTDTQHDKTKVIFDHVVKHERGIWKEMTAKYDPTGAWRKKYEEKA
KERGIVFPQD

>HmelCSP28
MASKIIVLLCCFAAMALAVEKYTDKFDNLDIQEILDNQRLLRSYANCLLDKGKCTPEGKE

MKEHIQDAIETGCEKCTDTQQDKTKVIFDHVIKHERGIWNEMTAKYDPTGAWRKKYEEKA
KERGIVFPQD

>HmelCSP29
MKIIVLFAFVATVVAVEFYTTGNDHLDMDALVNDKFELKYYVDCFLDKAPCTELTLSYKK
IIPESVKEACKKCNPNQRYQYWRFLEGLKAQLPEEYTNFRNYFDPENKYIDALENVVSKY
TKPDMDLLD

>HmelCSP30
MKIIVVFALIATVAAHEFYTTGNDRLDMDSLIANIPELQKFVDCFLERITCTELTTTYKV
ILEEAVKEACHKCNPNQKHQFWRFLEGLKVQLPEEYINFRHHYDPENKYFDALEKEISRY
HSRDMDLLDSIV

>HmelCSP31
MKAIVLLAFVAVAAADIEYYTTGNDHLDMNAVKADRMVLQAYMDCFNDRGPCTTLAESYK
RNIQESIVQACRRCNPNQKYMFWAGLQAAKELIPEDYWYFRHHYDPENKYFDAYEKEISK
YVKPDMEIVV

>HmelCSP32
MRSIVFFALFALVVADEFFKTGNDHLDVDAVLANRSELQAYLDCFEGKAPCTELAASYRR
NIPQAIVSACRLCNPNQKYIFWRFLQGLKAGYPEEYWNFRHFYDPEDIYFGGLERVISQY
ERPDMDSVQ

>HmelCSP33
MRIIVLFALFALAASDMMYYQSPNDHLDIDALLANHEELLSYLDCFEDKAPCSELLAAYK
RNIPEAIMTACRWCNPNQRYLFWKFLQGLKMFYPVEYWNFRHFYDPEDKYFEDLEQEISK
YTQPDMDIIM

>HmelCSP34
MRITVLFALFALVAADMMYYQSPNDHLDIDALLANHEELLSYLDCFEDKAPCSELLAAYK
KNIPEAVMTACRWCNPNQRYLFWKFLQGLKAFYPMEYWNFRHFYDPEDKYFEPLVQEISK
YTQPDMDIIM

>DPOGS208398-PA
MILKSCVVLCCVLVCVLGDEKYTDKYDNVNLQEILENKRLLEAYVNCILDKGKCSPEGKE
LKEHIQDALETGCEKCTDKQMEGTTTMIDHLVKHERAMWKELTDKFDPKGIWRKKYEDRA
REKGIVIPED-

>DPOGS208397-PA
MFVKMILKSCVVLCCVLVCVLGDEKYTDKYDNVNLQEILENKRLLEAYVNCVLDKGKCSP
EGKELKEHIQDALETGCEKCTDKQMEGTTTMIDHLVKHERAMWKELTDKFDPKGIWRKKY
EDRAREKGIVIPED-

>DPOGS210484-PA
MDLFPISVLLVACFTAINTQTYEKRYDSVNIDDVLSNKRLLAAYVKCVLDQGRCTPEGKE
LKSHIADALQSGCDKCTETQKDGVRKVIKHLIKNERDYWKQLVEKFDPEGVYAEKYEDEI
RDV-

>DPOGS208415-PA
MKSCVLLALLSLVAVAWGRPQHYTDKWDNINVDEILESQRLLRGYVDCLVDKGRCTSDAK
TLKETLPDALENDCKKCTEKQKTSADKVIRHLVNKRPDLWKELAGKYDPKDIYQQKYKNK
IEAVKEKH-

>DPOGS208414-PA
MKSCVLLALLSLVAVAWGRPQHYTDKWDNINVDEILESQRLLRGYVDCLVDKGRCTSDAK
TLKETLPDALENDCKKCTEKQKTSADKVIRHLVNKRPDLWKELAGKYDPKDIYQQKYKNK
IEAVKEKH-

>DPOGS208413-PA
MAVCYCRPDTYTDRYDSIDLDEILRNRRLLVPYIKCILEEGRCSADGKELKSHIKEALEN
DCAKCTKAQQDGTKKVIGHLINKENDYWQQLVEKYDPEHKYVEKYEKELRA-

>DPOGS208412-PA
MKSVGVFCLLSAVVLVTSRPETYTDRFDNINLQEIIDNHRLLKAYINCILDVGKCTNEGR
ELKMHIKDAIENRCDKCTEVQRNGTRIVIRHLINHEPSSWDELVQKYDPERKYVVKYEAE
LKDVLKD-

>DPOGS208400-PA
MKTLTPFSLFFMAKLCLGDNSYTTKYDGINLDEILASHRLLTGYINCLLDKGPCSPDGKE
LKNNLPEAIDNDCHKCTQRQKEGADKVMHFIIDNRPEDWDKLEEKYNSDGSYKLKYLTTK
LSENSEDVSKNYDEDDEEVNESRDHTSDNDHDTEMENH-
>DPOGS208394-PA
MRTFVVLACLVVSILAAEKYNSKYDNFDVETLVSNDRLLKSYINCFLEKGKCTAEGTDFK
KALPEAVETVCGKCTDKQKVNIRKVIKAIQQKHPTQWEELVKKNDPTGQHRANFEKFIQE
S-

>DPOGS210414-PA
MKTFVVFAMCLAAALALPADTYNPEYDNFNAKELVENPRLLKNYGKCFLDQGPCTAEGSD
FKKTIPEALRTTCAKCTPKQRELIRTVVRGFQKDLPEMWSELVKKEDPKGEYKESFEKFL
NGSD-

>DPOGS208396-PA
MKIFILLLCVFAAVIAEENSDLSTENIDLTEVVGNDKLIESYANCLINKGPCTPEMEKIK
EAIPEALETTCGKCSPKQKQLIRKVIKAIIEKQPQAWEQLVEKYDTEKKYRESFNKFIEE
EN-

>DPOGS214315-PA
MQLIVLCLCVCACVVGQDIEAMRNMPKYDSRYDYLDVDGLFNSKRLVKNYVECLVNGQRC
SPEGKALKRLLPEALRTKCVRCTERQKKIAVKIIKRLKIEYPDEWAKLQSKWDPTGDFTR
YFEVFLANEQFNSISGEGNEPSLPSLPSLPPLPPLPPLPPQTSSPQPATTAPTPPRPLLL
NRFADEVMEVSPSSAVTPMRPSTTRPTTSKPATVRPTTTRSPTMRQPVPPRPTPVSWANA
GSESMPTRFTLRPTKSELPLPYSTAITLIDQIGIKIIRTTELVTDILKNTVRAVVGR-

>DPOGS210485-PA
MKVVIILATLITLVLTQDTYSSDLENFDIDELLENDRLLESYGKCLEYKGPCTSQGREFR
KLMPEAIRTNCLKCTLKQREMVRKAAKALNVKLPKIWNELVKQEDPKGEFKQKFEDFLQR
SD-

>DPOGS208040-PA
MQLSHVIVIFAMAALCFAETQRPAVSDTALEDALNDKRFIQRQLKCALGEAPCDPIGKRL
KTLAPLVLRGACPQCSPQETKQIQRTLSYVQRNFPQQWAKIVRQYSG-

>DPOGS210162-PA
MYLALETSQLSKTKTLVALVAARPEDNYDRYENFDVDELVSNLRLLKSYAACFLGEGKCT
AEGNDFKKWIPEAVQSNCGKCSDHQKHLVGKVIKACIDKLPEEWNKLNAIHNPDGKYDEK
LKDLPGKIRKLNKMKFLVILALVALAAARPEANYEKYENFDVDELVSNLRLLKSYVACFI
GEGKCTPEGSDFKEWIPEAVQSNCGKCSDNQKHLVGKVIKACMEKLPEEWKKLNALHNPD
GKYDEGLKNFLDNYGH-

>DPOGS208418-PA
MILTVTCVLSYDEKYDRLDVDAILADDQHFTSYLDCFLDKAPCTTEYSKEFRELLPEVIK
TACEKCSDTQKVKVRKFVKAIFEKKAEMAQEFRTKFDPTGEYEPAFLAFLKQNK-

>DPOGS208393-PA
MKIVYFIFFIVFGVFADNKYYDSRYDYYDIDHFVQNPRLLKKYIDCFLDKGPCTPIGRVF
KLVLPEIVITACNKCSPSQKRFAHRTFEAFRNISPQNYAELRRKLDPQNKHFNTFLKSIA
TNS-

>DPOGS208399-PA
MDQGRCTPEGNTLKVHVTDAIQNSCSKCTEIQKTKARKVVNYIRENNKDVWDELIKKYDP
KDEYKEKYEAFLEGKF-

>DPOGS208392-PA
MKVIIVLSALLVLSSAETYNSENDDLDIEKLVSDPASLGAFLDCFNDKGACDELSGDFKK
DLREAVEQACEKCTAAQKHIFKRFLEVIKVQKADDYKIFQQKYDPENKYLPALEAAIAKY
-

>DPOGS209275-PA
MKTFILIALGALAAADPIDYYITDNTGLDIEALAAQPLKMISYINCFLDRGPCCRVGASY

KKNLAEAVSQACRRCSPDQKQLFWRFLHIIKPVFPQEYNEFWKKYDPEHIYYDLLMKELE
KYKPVSLPVLPIL-

>DPOGS208390-PA
MKIVILLSLLSVVAANDHFYYNLIPLEVTALAKNPKQIFEFLDCLLDKGPCNDVFEGYRD
IANQLVIYSLMAKAVYSSLHCSMAKKQSTTLPLLAIHPAPLKHVAFGNNPFLWKLSNRLV
KDVLPIKKDLNRYKMKIFITLAALVINAVVAEEELYDLPLLDMREIARNPDQLKSFLDCL
LDRAPCNPLYQGYRDLAPESIRESCRKCTPALKIFAYVFFTTLKTFLPEEYQNFRNKYDP
DNIYIDRLEEEVRKYAFGTFSG-

>DPOGS208388-PA
MKSVMILALFAFVAADKDYYVLDKIDLSKIENNIEELKIFMDCVLDKGTCSDLYNSYKVH
IDESFKTACGKCTPEQKQFVSQFFKLYRKVLPQDIEELINKYDPERKYIDDLLAEVDKYN
V-

>DPOGS208389-PA
MKLLFLLVLVSFAAAEEQFYSLQKIDLSKAEENIGEFKKFTDCLLEKGPCSDVYESYRVR
VNESLQSACGKCTPELKQFAAKFFEILKNYLPQEYDGFLKKYDPENKFDTTMKSIFLVFL
LAFVLNCAIADEQYYVLQKVNLSESSDIIGVMKNLMNCFLERSPCSEAFESYRVRIPEAF
QQACKKCSPEQKRFAAEFIQSLKAEMPEDYNDFIKKYDPENKYFDALEAELNKFI-

>DPOGS208391-PA
MKIVILLSLLSVVAANDHFYYNLIPLEVTALAKNPKQIFEFLDCLLDKGPCNDVFEGYRA
VSLEAVQQACKRCTADQKRFGNIFLMLLRKLLPQEYHNFRYKYDPKNKYFDALEAELSKY
KYLPCL-

>DPOGS208039-PA
MSDMQLERTLADRVMMQRHIKCALSEGPCDPTGMRLRTLAPLVLRGSCPQCSSQETRQIR
RTLAFVQRNYPWEWTKIVRQYG-

>DPOGS208395-PA
MDKGPCTKDGKNFKRVLPETLSTACGRCSPNQKIVVRKMLLGIRAKSEPRFFELLDKYDP
TRVNREALYSFLITGDERNF-

>DPOGS208419-PA
MKTIIFLAVIAFAVADPEFYNVATLDMDKMKNDPQEFKRLVDCLLDRQPCTPVYSTYRAI
VQEAIKKTCMKCSPIQKKAFWQFLQALRTIAPKDYDHFRQKLSSTMKTVIFLAAIAYVTA
AVPDYYDLVKLDMPKLSKDPKEFKIFVDCILDKGPCSPLYKTYRVAFAAANSQYYVLEKL
DMDLLVKNLDELKIFTNCLLEKIPCNKVHESYRVKTLEAVQEACKKCNPHTKHMFWEYLQ
ALKADLPKEYIEFRHKYDPDNKYFDIHEAEISKYAIE-

>DPOGS208387-PA
MKTIIFLAVIAFAVADPELYDVVTLDMDKMEKDPQEFNSLIDCLLDRGPCTPVFQTYRGI

VQEATENICKKCNPYQKRAYWHFLRILRTTNPVDYENFRQKFDADNVYVDILESVLRGFA
NLNQMHVPQTTNVSLVAPLIKETRKELIIVMHYVRFFCNMFRGFILPQLLWDEVNLPIVV
RLSNALIVLQPTVIAFAVADPELYDVVTLDMDKMEKDPQEFNSLIDCLLDRGPCTPVFQT
YRGIVQEATENICKKCNPYQKRAYWHFLRILRTTNPVDYENFRQKFDADNVYVDILESVL
RGFANLK-

>DPOGS209276-PA
MKSIVLFAVIAIAFAEYPYYNLVKLDMDHISKDPLELRRLSDCLLDRGPCTPVYDTYKAV
TKEAIESLCMKCSLYQKHIFWQFLQALRAISPNDYILYKQKYDVDNKYFGILESILSQFN
DSY-

>DPOGS208410-PA
MFATLSVINTEMVVPGIDRTMSEGVTSIGYKVVYGDEDLDVINEVVSDSEKLETFKKNSE
LNKLLPPLPSIDVKCLMSVDRYCSKDMGEMKSILIQALKDDCAKCTQEQKESAGKIVAAM
MAHDPVAWKQFLTRSALEILPKKKPPKEVKHIFKQVGEPEYIENSKHRYTTPGVKVRVKR
YATKRVN-

>DPOGS204592-PA
MKFSVILALVALVAAPPEDNYDRYENFDVDELASNLQWIPDAVQSNCGKFSENQKHIIGR
VNKAFIDKLPEEWKKLKAIHNPGGKYVEGPKNVLDKYE-

>DPOGS208411-PA
MRRSFVTYLILIVTCETISVKGQRDAQNYTNRLIYNDGNLIVLNDVILSEDCLAEWNDDI
DQNKFIPPIGIKQIKCLMSVDRHCTKSMAHVKGILIKALKDGCEECSIKENESVGKIIAS
MWAYDTDTWKMFLTKYNSMDILQRIF-

>DPOGS210415-PA
MKTFILIALGALAAADPIDYYITDNTGLDIEALAAQPLKMISYINCFLDRGPCCREYNEF
WKKYDPEHIYYDLLMKELEKYKPVSLPVLPIL-

>PxylCSP1
MKAAAFIALFLIGKAVCEDKPTYTTKYDNIDLDEILSSERLLTGYVNCLLDQGPCTPDGK
ELKHTLPDAIDNDCRKCTQKQKEGSDRVMGYIIEYRPNDWAKLEKKYLSDGSYKKKYLEK
KNASENNGDSKSTEAKNKDDEEKKGKGDGEEK

>PxylCSP2
MQKLTLACLLVAVAAAAARPNDSHYTDRYDNVNLDELISNRRLLVPYVKCVLDQGKCSPD
GKELKEHIQEALENNCGKCTDKQREGTRKMIGHLINHEQEFWDQLIAKYDPERKYVSKYE
KELKEVKA

>PxylCSP3
MNSLVLVCLALAAVAAARPQATYTSKYDGVNVDEILANDRLMMPYIKCALDHGRCSPEAK
ELKSHIKEALENNCAKCTDKQKPAVRKVIAHLINHKPAEWRQLSDKYDPAGKYTAQYEDQ

LR

>PxylCSP4
MQTVTLLCLLAAVAAAAAAPADTYDAKYDSFNAHELVQNQRLLKSYGKCFLSKGPCTAEG
SDFKRVIPEALKTTCGKCTRKQRELVRVVVKGFQEQLPQVWTEIVSKEDPKGEYKDSFAK
FLEGSD

>PxylCSP5
MTSLKLLALACVMAAALADDTYTDKYDNINLQEILDNKRLLMNYVNCVLEKGKCNAEGKE
LKDHLEEALQTGCAKCTEAQKKGAQTVIEHLIKNELELWRELANKYDPQGTFRKKYEDQA
KEHGIVIPDE

>PxylCSP6
MRVLMLVALSCAVGAAWAKPASTYTDKFDNVNLDEILESNRLLKGYVDCLLDKGRCTPDA
KTLKETLPDALENDCSKCTEKQKSGSDKVIRHLINKRPELWKELAVRYDPNNIYQDKFKD
KIEAVKTVKA

>PxylCSP7
MTIWALLAVVALASAALAQQQEYPTNTKYENFNTDSIIANERILLGYYKCVMDKGPCTKD
GKNFKRVLPLTITTACSHCSPKQKTVVRRLLLGIRAKSEARFMEMLDKYDPGRLNRPALF
TFLANGN

>PxylCSP8
MKLFVALCFVTLVAYSSARPNGSYTDRYDNLDLDEILNNSRLRVPYVKCLLGKGKCSPDG
KELKSHVREALENQCGKCTPAQQAGTRKVIGYLINNEAGYWQELVALYDPQRKYVKQYET
ELRKVSG

>PxylCSP9
MKLLIITLALAATCVAAQKRYTNKYDNMDLDEVLANRRLLVSYIKCVLDQGRCTAEGKEL
KTHISDALQTGCKKCTPNQREGARRVISHMIKEEPEYWTMLVEKYDPERMYSTKYEKEIN
SIQ

>PxylCSP10
MNSLLLFVCLSLAGLAASEQYTDRYDNLNVEEILSIRRLLVPYIKCMLEQGRCTPEGKEL
KLHVKDGMQTGCSKCTPWQRTNARKVVKHIREKETEYWEALKKKYDPKDEFKPTYEAFLA
ADD

>PxylCSP11
MRSLIILCCLAAGALAADKYNPKYDNFDVETLISNDRLLKSYINCFLEKGRCTPEGSDFR
KALPEAVETICAKCTEKQKTNIKKVIKAIQQRHPKQWEELVKKNDPTGNHRDQFEKFIHD
KS

>PxylCSP12

MKVAIIVSMLVVAACAQDKYKSDLEGDFDVTELLNNERLLLSYTRCLIDKGPCTPEVKAV
KDKLPEALATKCAKCTDKQKELGKKLAVELKRTHPAVWAQLVAKYDPQGQHQAAFQEFLT
KQ

>PxylCSP13
MKLLLLSLSLLALSQGAPYSAEHDDFDVEPVLRDPALLKSFSDCFLDKGPCDDIQSHFKK
NIPDAVQTACSSCTDKQKHLMRRVLEVLTVQYPDVAEQFTTKYDPEKKYIPALLAAVDKA

>PxylCSP14
MQFSITHLLVLSALVAVCWAQAKETKPRVSESALEEALNDKRYIQRQLKCALGEAPCDPT
GKRLKTLAPLVLRGACPQCTPQETKQIQRTLSYVQRNFPQEWAKIVRQYAG

>PxylCSP15
MRRILAAALAAGLIALASTAVDLNNLEDMDVEALFRDKARSAAAFQCLMDQGPCGELQPL
RDSLPSMVETQCANCSPKQREKYVQVNLLLLAQYPAEYEKLALKYSPKLD

>HassCSP1
MKFLLVLCMLAAAALADDKYTDKYDNINLDEILENKRLLIAYVNCVMERGKCSPEGKELKEHLQDAIE
TGCTKCTEAQEKGAYKVIEHLIKNELDIWRELTAKYDPKGDWRKKYEDRARANGIQIPE*

>HassCSP2
MKVVLLTLCFALGVLAQDQYEAANDNFDISEVIGNDRLLHAYANCLLNKGPCTPEVKQVKEKLPEALE
TRCAKCTDKQKQMGKALAQEVKKNHPDIWKQLVAMYDPQGKYQQAWKDFLQE*

>HassCSP4
MMKYIALAMMLLVYLTIQSNAVETSTYTTKYDGIDLDEILNNERLLTGYVNCLMDNGPCTADGKELKR
NLPDAIENDCKKCTDRQREGADRVMHYLIDHRPEDWTKLEKKYNSDGSYKMKYLSRKSAEDSKETNAT
KSDEDTKNETKEST*

>HassCSP5
MKFLVVAIVVTLAAFAVAEKYTDRYDHIDIDEIIANRKLLVPYIKCVLDQGRCTPEGRELRVNIKDAL
QTSCTKCTAKQKKASRKVIKHIRTHEDDYWKQLLAKYDPDTVYVKNYESFLAAED*

>HassCSP6
MKVFVVLSVLIAFTAAASLTPAELDMAEAFDYEALFSNDEQRKIVFNCMLGKGECGDYQKLAEISRKI
LESKCADCSPSQKAKYETVLKTIQTKYEPFYNELLAAKKE*

>HassCSP7
MQVVVFLSVICVGLVAGLHVQAGPQMTDAQLDQTLADKNTMQRHIKCALGEGPCDPVGRRLRTLAPLV
LRGACPQCSMQETRQIRRTLAFVQRNYPWEWAKIVRQYG*

>HassCSP8
MWTQVITFSVALTIASGEEVAGGIKREVSEGVQSMGYKLIYGDEDMTIINQVVTDAEKSSMIKKNKVN
LNEAIKPLAPEDVKCLMSVDRYCSKEMGAMKSVLIQAVKEDCAKCSTKQKDDAGKVIASMMAHDPVAW
KLFLTRYDGLHKVQRILG*

>HassCSP9

MRTFVVVCLLGLVAVTLARPDSKYTSKYDNINLDEILANQRLLVPYLKCILEEGKCTPEGKELKSHIR
EALEEDCAKCTENQRKGTRKVLAHLINHEEGYWNRLKAKYDPESKYTSKHEQDLRELKH*

>HassCSP10

MKILVLLLAAVVTAQYEEDTYGTDHDDLNIAAVVEDKEQFNSFVDCFIDEAPCDEVADTFKSVIPEAV
LEACAKCTPAQKHIVRVFNESFKKKMPEKFQKFKNKYDPEGKYFDNFEAAVAAF*

>HassCSP11

MKSVLVLCLVIAAVWARPETYDTRYDDFDAETLVENVRLLKAYGHCFLGTGPCTPEGSDFKKTIPDAL
RTGCGKCTAKQRHLIRVVVQGFQSKTPDLWQQLVKKEDPNGQYKEIFTRFLNGSD*

>HassCSP12

MKCIYVLSFLLALAAVQAEDKYSTENDNLDIDAVVANVDSLRNFVACFLDEEPCDAVAADFKKDIPEA
VTTSCAKCTDAQKHIFYKFLLGLKKKLPKQYEEFRRKFDPENKHFSALENAVSPA*

>HassCSP13

MKLLIVLALVAVVAARPDDAFYDKKYDDFNVDEIIENVRLLKAYAHCIIGDGKCTPEGNDFKRWVPEA
TKSSCGKCTEKQKVLVAKTIKAIQEKCPEEYTTLVKQIDPENKYAEDLKNYLAKYGH*

>HassCSP14

MKLIIAVALLCLVAESWAASTYTDKWDNINVDEILESQRLLKAYVDCLLDRGRCTPDGKALKETLPDA
LENECSKCTDKQKSGSDKVIRHLVNKRPELWKELSAKYDPNNIYQDRYKDKIEAVKGQ*

>HassCSP15p

MKADCFLIVTLVAVVAADFYNSKYDSFDVQPLLENDRILLGYTKCFLDQGPCTPDAKDFKKVIPEALE
TTCGKCSPKQKQLIKTVIKAVISRHPDAWDQLIEKYDKDKKY

>HassCSP17

MRSWLICLCVLTVVVSCYSQAPNRYENFNADAIIQNDRILLAYYKCVMDKGPCTRDGKNFKRVLPETL
ATACGRCNPKQKTIVRKLLLGIRSKSEPRFLELLDKYNPDRSNRDALYAFLVTGN*

>HassCSP18

MKVLIVAVLALVAPSALGYDEKYDKLDVDKIIGDDALFTAYTDCMLDKGPCTVEHSEDFKKLLPEVIQ
TACAKCSGIQKTNVRKTVKALSDKKPDDFAKFRAKFDPKGEYEKDFSAFMLATD*

>HassCSP19

MIDLNKCNFRLLISILCLFFVSINAQQRFYDRRYDYYDIDTLIQNPRLLKKYLDCFLEKGPCTPIGRV
FRQLLPDAVATACEKCSPSQRRLARKTFNAIRRNFPQGYVELMSKLDPKNKYYEAFEKAIANA*

>HassCSP20

MKAVFLLCLVVVAVSARPEAQYTNKYDNVNLDEILVNKRLLVPYIKCALDQGKCSPDGRELKSHIQEA
LENYCAKCTPVQQDGTRRVIAHLINHEPEYWRQLSAKYDRDGKYAARYENELRTIA*

>HassCSP23

MNSAIVLCVVALAGMVLARPDDTYTTKYDNVDLDEILANDRLLVPYIKCVLDEGKCAPDAKELKEHIR
EALENGCAKCTDKQKEGTRRVIAHLIKHKPEEWEKLKAKYDPEGKYTHKYEKELEEVKKA*

>HassCSP26

MNSLIVFCVLSLAALTIARPDGATYTDKYDNVDLDEILGNRRLMVPYIKCMLDQGKCAPDAKELKEHI
REALENECGKCTEAQKNGTRRVIGHLINHEADYWNELTAKYDPERKYTTKYEKELKEVKA*

>HassCSP27

IREALENACGKCTAAQQSGTRRVIGHLINKEPEFWKQLNAKYDPNNKYTKKYEKELKEVQAENQ*

>HassCSP28

MKFILIAMAIALVCVSAEEYPNKYDNMNLDEVLGNKRLLNGYMKCALDKGPCTAEGRDLKYYISDGLK
TGCSKCTDRQRKGIKKVMKHLIKHEPEYWKQAVDKYDPDRLYTKMYEKEVESWS*

>HzeaCSP8

MWTQVIIFSVALTIAIGEEVVPGIKREVSEGVQSMGYKLIYGDEDMTIINQVVTDAEKSDMVKKNKVN
LNEAIKPLAPEDVKCLMSVDRYCSKKMGAMKSVLIQAVKEDCAKCSIKQKDEAGKVIASMMAHDPVAW
KLFLTRYDGLDKVQRILG

>HzeaCSP4

MMLLVYLTIQSNAVETSTYTTKYDGIDLDEILNNERLLTGYVNCLMDNGPCTADGKELKRNLPDAIEN
DCKKCTDRQREGADRVMHYLIDHRPEDWTKLEKKYNSDGSYKMKYLSRKPAEDSKETNTTKSEEDTKN
ETKEST

>HzeaCSP9

MRTFVVVCLLGLVAVTLARPESKYTSKYDNINLDEILANQRLLVPYLKCILEEGKCTPEGKELKSHIR
EALEEDCAKCTENQRKGTRKVLAHLINHEEGYWNRLKAKYDPESKYTAKHEQELRELKH

>HzeaCSP25

MNSAIVLCVVALAGMVLARPDGDGDKYTSRWDDVDLDEILENDHLLVPYIKCSLDEGKCAPDAKELKE
HIQEALETGCAKCTDKQKEGTRRVIAHLIKKKLQEWEKLKAKYDPEGKYAKKYEKELEEVKNA

>HzeaCSP24

MNSAIVLCVVALAGMVLARPDGDGDKYTSRWDNIDLDEILGNDRLLVPYIKCALDEGKCAPDAKELKE
HILEALETGCDKCTDKQKEGTRRVIAHLIKYKLEEWEKLRAKYDSEGKYAKKYEKELEELKNA

>HzeaCSP27

MNSLIVFCVLFLAALTIARPDGATYTDKYDNIDLDEVLSNRRLLVPYVHCLLEQGKCAPDAKELKEHI
REALENACGKCTNAQQSGTRRVIGHLINKEPEFWKQLNAKYDPNNKYTKKYEKELKEVQEEHH

>HzeaCSP23

MNSAIVLCVVALAGMVLARPDDTYTTKYDNVDLDEILGNDRLLVPYIKCTLDEGKCAPDAKELKEHIR
EALENGCAKCTDKQKEGTRRVIAHLIKHKLEEWEKLKAKYDPEGKYTHKYEKELEEVKNA

>HzeaCSP22

MNSAIVLCLVALAGMVLARPDGGTYTTKYDNVDLDEILANDRLLIPYIKCLLDEGKCAPDAKELKEHI
REALENGCAKCTDKQKEGTRRVIAHLIKHKNADWQKLKAKYDPEGKYTHKYEKELEEVQH

>HzeaCSP21

MNSAIVLCLVALAGMVLARPDGGTYTTKYDNVDLDEILANDRLLIPYIKCLLDEGKCAPDAKELKEHI
REALENGCAKCTDKQKEGTRRVIAHLIKHKNADWQKLKAKYDPEGKYTHKYEKELEEVQH

>HzeaCSP26

MNSLIVFCVLSLAALTIARPDGATYTDKYDNVDLDEILGNRRLMVPYIKCMLDQGKCAPDAKELKEHI
KEALENECGKCTEAQKKGTRRVIGHLINHEADFWNELTAKYDPERKYTTKYEKELKEVKA

>HzeaCSP20

MKAVFLLCLVVVAVSARPEAQYTNKYDNVNLDEILVNKRLLVPYIKCALDQGKCSPDGRELKSHIREA
LENYCAKCTPVQQDGTRRVIAHLINHEPDYWRQLSVKYDRDGKFAVKYEKELRTIA

>HzeaCSP5

MKFVVVALVVTLAAFAVAETYTDRYDNINIDEIIENRKLLVPYIKCVLEQGRCTPEGRELKAHIKDAL
QTSCTKCTQKQRKASRKVVKHIRANELDYWKQLLAKYDPDNVYVKNYESFLAADD

>HzeaCSP1

MKVLLVLCLVAAAALADDKYTDKYDNINLDEILENKRLLLAYVNCVMERGKCSPEGKELKEHLQDAIE
TGCSKCTEAQEKGAYKVIEHLIKNELDIWRELAAKYDPKGDWRKKYEDRARANGIQIPE

>HzeaCSP14

MKLIVAVALLCLVAESWAASTYTDKWDNINVDEILESQRLLKAYVDCLLDRGRCTPDGKALKETLPDA
LENECSKCTDKQKSGSDKVIRHLVNKRPEMWKELSAKYDPNNIYQDRYKDKIEAVKGQ

>HzeaCSP15

MKADCFLIITLVAVVSADFYNSKYDSFDVQPLLENDRILLSYTKCFLDQGPCTPDAKDFKKVIPEALE
TTCGRCSPKQKQLIKTVIKAVISRHPDAWDQLTEKYDKDQKYKESFDKFLAEQD

>HzeaCSP2

MKVVLLTLCFALGVLAQDQYESANDNFDISEVIGNDRLLHAYANCLLNKGPCTPEVKQVKEKLPEALE
TRCAKCTDKQKQMGKALAQEVKKNHPDIWKQLVAMYDPQGKYQQAWKDFLQE

>HzeaCSP17

MRSWLICLCVLTVVVTCHSQAPNRYENFNADAIIQNDRILLAYYKCVMDKGPCTRDGKNFKRVLPETL
ATACGRCNPKQKTIVRKLLLGIRSKSEPRFLELLDKYNPDRSNRDALYAFLVTGN

>HzeaCSP6

MKVFVVLSVLIAFTAAASLTPAELDLVEAFDYEALFSNDEQRKLVFDCMLGKGDCGDYQKMAEISRKV
LESKCADCSPSQKAKYETVLKTIQTKYEPFYNELLKNVAAKKE

>HzeaCSP18

MNVLLVAVLALVAPSALGYDEKYDKLDVDKIIGDDALFTAYIDCMLDKGPCTVEHSEDFKKLLPEVIQ
TACAKCSGIQRTNVRKTVKALSDKKPDDFAKFRAKFDPKGEYEKDFSAFMLGTD

>HzeaCSP3

MKVLVVLSCLIVAAFAADKYNAKYDNFDVDTLITNDRLLKAYINCFLDKGRCTPEGSDFKKTLPEAIE
TTCGKCTDKQKNNIRKVIKAIQQKHPKEWDALVKKNDPSGKHRANFDKFIQGSR

>HzeaCSP19

MIDLNKCNLKVSILILCLFFVSINAQQRFYDRRYDYYDIDTLIQNPRLLKKYLDCFLEKGPCTPIGRV
FRQILPDAVATACEKCSPSQRRLARKTFNAIRRNFPQGYVELMSKLDPKNKYYEAFEKAIANA

>HzeaCSP12

MKCIYVLSFLLALAAVQAEDKYSTENDNLDIDAVVANVDSLTNFVACFLDQEPCDAVAADFKKDIPEA
VTTRCAKCTDAQKHIFYKFILGLKEELPRGYEEFRRKYDPENKHFSALENAVSPA

>HzeaCSP10

MKILVLLLAAVVIAQYEEDTYGTDHDDLDIVSLVEDKDQFNSFVDCFIDEAPCDDVAETFKSVIPEAV
LEACAKCTPAQKHIVRVFNESFKKKIPEKFQKFKNKYDPEGKYFDSFDAAVAAF

>HzeaCSP11

MKSLLVLCLVIAAVWARPETYDTRYDDFDAETLVENVRLLKAYGHCFLGTGPCTPEGSDFKKTIPDAL
RTGCGKCTAKQRHLIRVVVQGFQSKTPDLWQQLVKKEDPNGQYKEIFTRFLNGSD

>HzeaCSP13

MKLLIVLALVAVVAARPDDEFYDKKYDDFNVDEIIENVRLLKAYAHCIIGDGKCTPEGNDFKRWVPEA
TKSSCGKCTEKQKVLVAKTIKAIKEKCPEEYTTLIKQLDPENKYAEDLKNYLAKYGH

>HzeaCSP16

MQTRYAVVLCCVVAACVAQTQRPPVSDSALEDALQDKRFIQRQLKCALGEAPCDPIGKRLKTLAPLVL
RGACPQCTPQETKQIQRTLSYVQRNFPQQWAKIVRQYAG

>HzeaCSP7

MQVVVFLSVICVGLVAGLHVQAGPQMTDAQLDQTLADKNTMQRHIKCALGEGPCDPVGRRLRTLAPLV
LRGACPQCSMQETRQIRRTLAFVQRNYPWEWAKIVRQYG
