# Supplementary figures and images for: Chemosensory Proteins (CSPs) in the Cotton Bollworm Helicoverpa armigera
Source: Insects. 2021 Dec 27;13(1):29. doi: 10.3390/insects13010029 (PMC8780252; doi:10.3390/insects13010029)

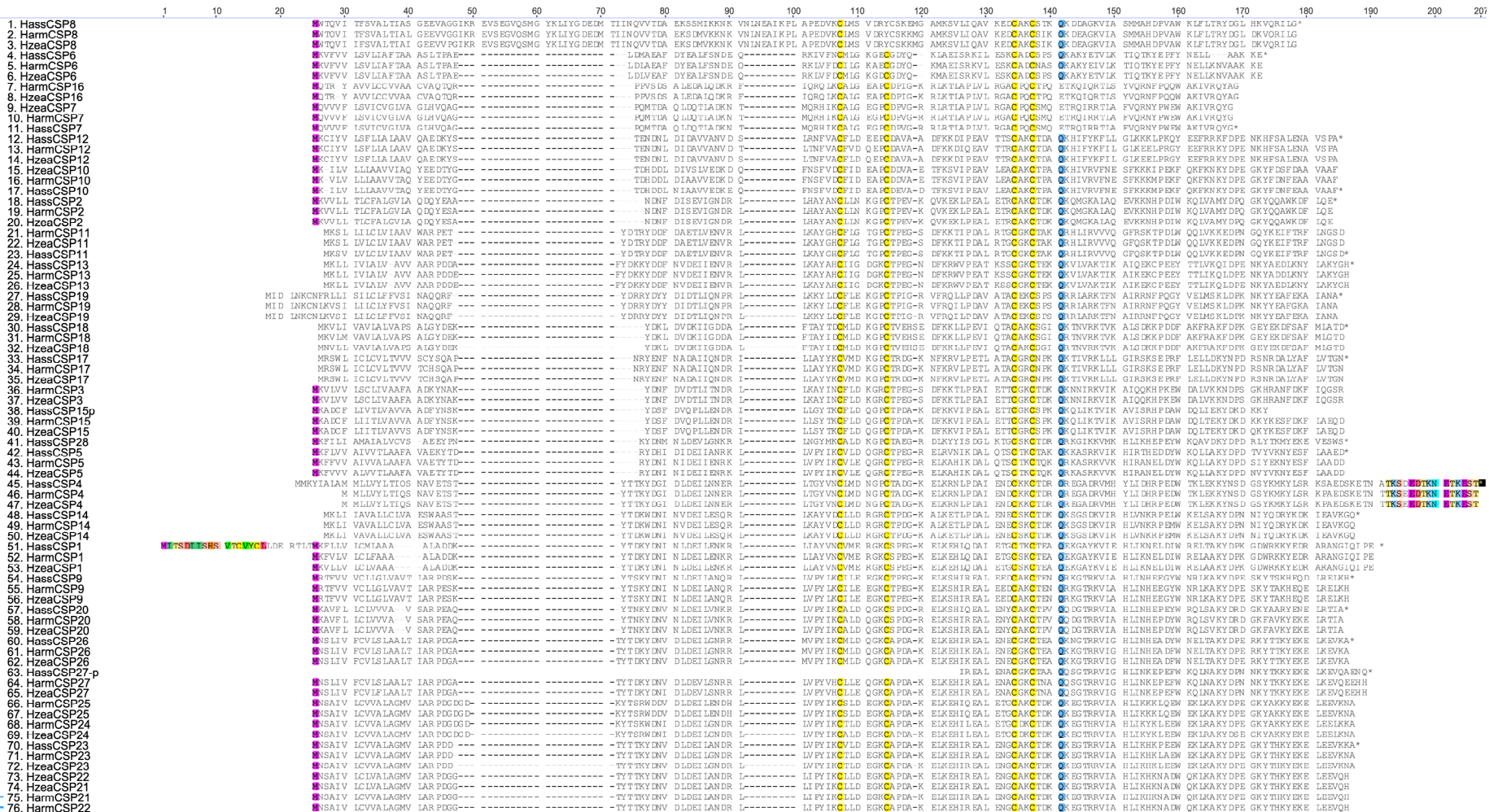

Figure S1. The alignment of HarmCSPs, HzeaCSPs, and HassCSPs.

Supplement: Supplementary file 1 [file insects-13-00029-s001.zip › Figure S1.pdf]
